# Multistate outbreak of *Salmonella* Mbandaka infections linked to sweetened puffed wheat cereal – United States, 2018

**DOI:** 10.1017/S095026882200108X

**Published:** 2022-06-20

**Authors:** Amelia A. Keaton, Colin A. Schwensohn, Joshua M. Brandenburg, Evelyn Pereira, Brandon Adcock, Selam Tecle, Rachel Hinnenkamp, Jeff Havens, Kim Bailey, Brad Applegate, Pamela Whitney, Deborah Gibson, Kathy Manion, Michelle Griffin, Joy Ritter, Carrie Biskupiak, Kadri Ajileye, Mugdha Golwalkar, Michael Gosciminski, Brendalee Viveiros, Genevieve Caron, Laine McCullough, Lori Smith, Eshaw Vidyaprakash, Matthew Doyle, Cerise Hardy, Elisa L. Elliot, Laura B. Gieraltowski

**Affiliations:** 1 Epidemic Intelligence Service, Centers for Disease Control and Prevention (CDC), Atlanta, GA, USA; 2 Division of Foodborne, Waterborne, and Environmental Diseases, National Center for Emerging and Zoonotic Infectious Diseases, CDC, Atlanta, GA, USA; 3 Coordinated Outbreak Response and Evaluation Network, Food and Drug Administration (FDA), College Park, MD, USA; 4 California Department of Public Health, Richmond, CA, USA; 5 Montana Department of Public Health and Human Services, Helena, Montana, USA; 6 RiverStone Health, Billings, MT, USA; 7 Missoula City-County Health Department, Missoula, MT, USA; 8 New York State Department of Health, Albany, NY, USA; 9 Tennessee Department of Health; 10 Rhode Island Department of Health; 11 Utah Department of Health

**Keywords:** Food safety, food-borne infections, salmonella, *Salmonella enterica*, salmonellosis

## Abstract

In May of 2018, PulseNet, the national molecular subtyping network for enteric pathogens, detected a multistate cluster of illnesses caused by an uncommon molecular subtype of *Salmonella* serovar Mbandaka. A case was defined as an illness in a person infected with the outbreak strain of Salmonella Mbandaka with illness onset on or after 3 March 2018 and before 1 September 2018. One-hundred thirty-six cases from 36 states were identified; 35 hospitalisations and no deaths were reported. Ill people ranged in age from <1 year to 95 years (median: 57 years). When standardised questionnaires did not generate a strong hypothesis, opened-ended interviews were performed. Sixty-three of 84 (75%) ultimately reported consuming or possibly consuming a specific sweetened puffed wheat cereal in the week before illness onset. Environmental sampling performed at the cereal manufacturing facility yielded the outbreak strain. The outbreak strain was also isolated from open cereal samples from ill people's homes and from a sealed retail sample. Due to these findings, the brand owner of the product issued a voluntary recall of the cereal on 14 June 2018. Additional investigation of the manufacturing facility identified persistent environmental contamination with *Salmonella* Mbandaka that was closely genetically related to other isolates in the outbreak. This investigation highlights the ability of *Salmonella* to survive in low-moisture environments, and the potential for prolonged outbreaks linked to products with long shelf lives and large distribution areas.

## Introduction

Salmonellosis is among the most common foodborne illnesses and accounts for over one million illnesses per year in the United States [1]. Although *Salmonella enterica* most commonly results in a self-limited diarrhoeal illness, children and older adults are at higher risk for complications such as dehydration, bacteremia and infection of other sites outside of the gastrointestinal tract. Most *Salmonella* serovars are known to infect and colonise the intestines of animals, and outbreaks of human infections have historically been linked to consumption of contaminated meat products or fresh produce items. For example, from 2009–2015, the most commonly reported confirmed food vehicles for human *Salmonella* outbreaks were eggs, chicken and pork [2]. The investigation of foodborne *Salmonella* outbreaks, in turn, has focused on these categories of food items.

Despite the relative frequency of meat- and produce-associated *Salmonella* outbreaks, multiple outbreaks within the United States (US) have demonstrated the ability of *Salmonella* to contaminate and survive in a wide variety of products, including dry, shelf-stable nut and seed products, nutritional powders, spices and kratom (a dried leaf powder used in teas and capsules as a stimulant and/or opioid substitute) within the past decade [3–6]. Such outbreak vehicles are often not initially suspected by either the people who are sick or public health investigators, as many assume that such items have undergone a processing or ‘kill’ step that would eliminate pathogens such as *Salmonella.* Here, we describe a multistate outbreak of a relatively uncommon *Salmonella* serovar, *Salmonella* Mbandaka, that was ultimately linked to a ready-to-eat (RTE) breakfast cereal product.

## Methods

### Detection of outbreak

On 30 April 2018, epidemiologists at the Centers for Disease Control and Prevention (CDC) received notification by PulseNet of 12 *Salmonella* Mbandaka infections from 11 different states. PulseNet is the national molecular subtyping network and database established by the CDC for the surveillance of foodborne, waterborne and zoonotic pathogens. Because salmonellosis is a nationally notifiable disease in the United States, any available *Salmonella* isolates collected from clinical specimens are submitted to PulseNet's network of laboratories for further molecular subtyping through either pulsed-field gel electrophoresis (PFGE) or whole-genome sequencing (WGS). Isolates with indistinguishable PFGE patterns or that are highly related by WGS are more likely to share a common source [8]. Bacterial isolates collected by regulatory agencies during routine inspections of food or and agricultural facilities are also uploaded to PulseNet for molecular subtyping to aide foodborne, waterborne or zoonotic pathogen investigations. Database analysts then notify public health officials if the number of isolates of particular molecular subtype is higher than expected for a particular month, thus allowing for detection of potential outbreaks that may otherwise have gone undetected [7]. Isolates with indistinguishable PFGE patterns or that are highly related by WGS are more likely to share a common source [8]. All 12 Salmonella Mbandaka isolates reported to CDC shared an indistinguishable pulsed-field gel electrophoresis (PFGE) pattern (*Xba*I restriction enzyme pattern TDRX01.0004). This PFGE pattern is considered rare and had been identified in a total of 41 individual isolates since the creation of PulseNet in 1996; while most isolates were from human clinical specimens, 3 were from different food samples that had not been linked to any human illnesses. Further, no prior outbreaks of this *Salmonella* PFGE type had been reported to CDC, suggesting that prior clinical specimens were collected from sporadic cases of salmonellosis. CDC, the United States Food and Drug Administration (FDA), and state and local health departments began an investigation to determine the scope and severity of this outbreak, to determine a potential source and implement measures to limit the spread of illness.

### Case definition and case finding

A case was defined as infection in a person within the United States with the outbreak strain of *Salmonella enterica* serovar Mbandaka with illness onset on or after 3 March 2018 and before 1 September 2018. The outbreak strain was initially defined as an isolate matching PFGE *Xba*I pattern TDRX01.0004; however, CDC and state laboratories performed whole genome sequencing (WGS) analysis, which was later incorporated into the case definition. The use of WGS and the PulseNet database also identified multiple isolates matching *XbaI* enzyme pattern TDRX01.0005 that were highly genetically related to case isolates with *XbaI* pattern TDRX01.0004, and so this second enzyme pattern was later incorporated into the outbreak case definition. The use of PulseNet allowed for detection and inclusion of any new cases detected during the outbreak

### Hypothesis generation

State and local health officials first interviewed case-patients using state-specific questionnaires to collect exposure information. Once CDC confirmed that this strain of *Salmonella* Mbandaka had been identified from case-patients in multiple states, state and local public health officials employed the National Hypothesis Generating Questionnaire (NHGQ), a standard questionnaire utilised by public health officials to rapidly collect information regarding food, animal and environmental exposures during the seven days preceding illness. Frequencies of exposures identified using this questionnaire were then compared to responses to the 2006–2007 Foodborne Diseases Active Surveillance Network (FoodNet) Population Survey using a binomial probability distribution. The FoodNet survey compiles data from interviews of healthy people nationwide that include questions about foods they ate during the seven days before interview [9].

As analysis of NHGQs did not generate strong hypotheses as to the cause of the outbreak, we conducted open-ended narrative interviews with a subset of recently reported case-patients to obtain a more in-depth food history for the week before illness onset. Whereas NHGQ questionnaires collect structured answers to a variety of food exposures, open-ended interviews allow respondents to discuss normal routines and habits that may reveal previously undocumented exposures. Results from these interviews were then used to develop focused questions for state and local health departments to interview newly identified case-patients to gather more specific information on foods and brands of interest.

### Regulatory investigation

State and local health departments collected detailed product information, including point of sale locations, purchase dates, product photos and lot codes during interviews with ill people.

FDA conducted a comprehensive inspection to assess the identified manufacturing facility's compliance with the Current Good Manufacturing Practice, Hazard Analysis and Risk-Based Preventive Controls for Human Food regulation. This included a review of production processes and review and collection of documents related to the manufacturer's employee training, food safety plan, process controls, allergen programme, supply chain programme, sanitation controls and environmental monitoring programme. Distribution records, microbial testing records and various production records were collected. Environmental sampling was conducted throughout the manufacturing environment.

### Laboratory investigation

FDA collected and processed samples for microbiologic testing during the comprehensive facility inspection. Serotyping and PFGE and/or WGS were performed by FDA utilising methods described previously [6].

State and local public health officials collected open product samples from the homes of ill people. Retail samples of the cereal were also collected for microbiologic testing at state public health laboratories. State public health laboratories serotyped and subtyped any clinical *Salmonella* isolates identified by PFGE using standard methodology [10]. Following PulseNet protocols, public health laboratories further characterised a sub-set of clinical and cereal isolates by WGS. Genomic DNA was extracted using QIAGEN DNeasy Blood and Tissue Kit (Qiagen, Aarhus, Denmark). DNA libraries were prepared using Nextera XT DNA Library Preparation Kit (Illumina, San Diego, CA), and the DNA sequencing was performed on the Illumina MiSeq Sequencing System using the 2 × 250 base pair sequencing chemistry. High-quality single nucleotide polymorphism (hqSNP) analysis was performed using the Lyve-SET pipeline for both isolates collected by public health and FDA laboratories. Depending on the serotype, either closed PacBio sequences or draft Illumina assemblies were used as references with prophages removed from the analysis. Read mapping was performed using sequencing mapping and alignment, and SNPs were called using VarScan at >20x coverage, >95% read support, and clustered SNPs <5 base pair apart were filtered out.

## Results

**Fig. 1.**
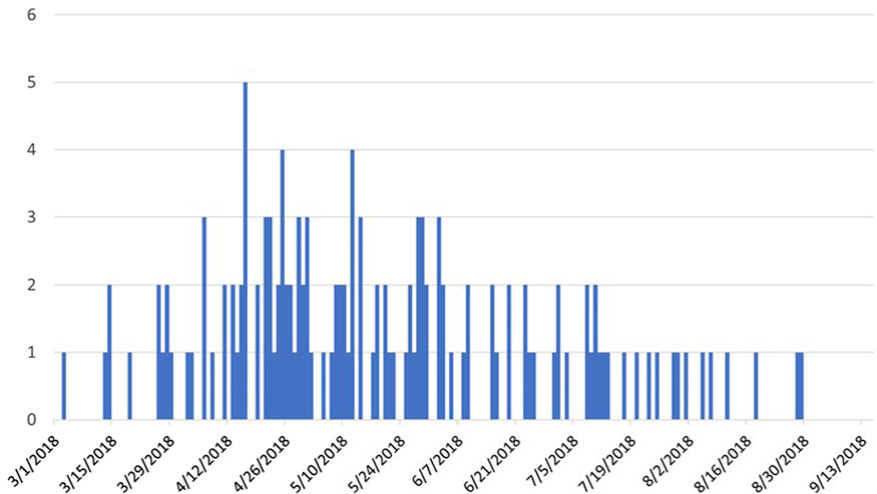
People infected with the outbreak strain(s) of *Salmonella* Mbandaka by date of illness onset (*n* = 136), United States, March–September 2018.

**Fig. 2.**
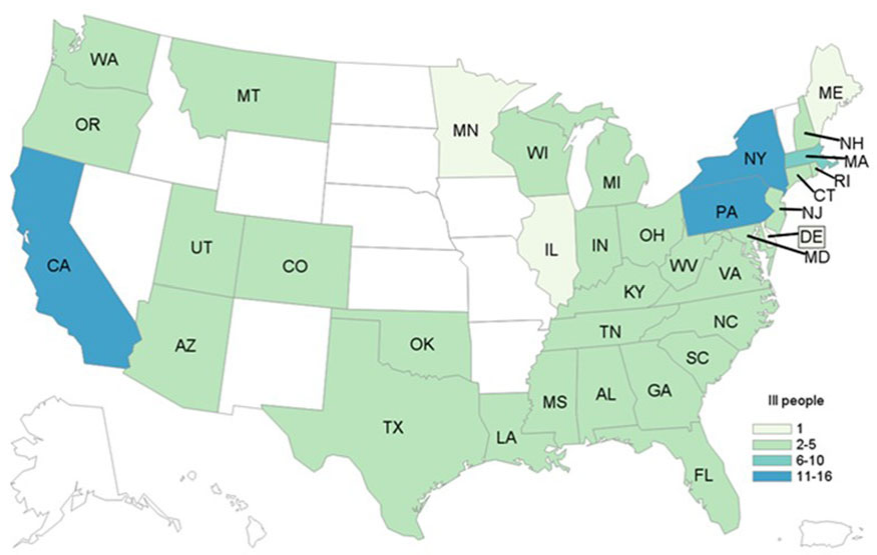
People infected with the outbreak strain(s) of *Salmonella* Mbandaka by state of residence (*n* = 136), United States, March–September 2018.

We identified 136 cases from 36 states during the investigation (Fig. 1). Illness onset dates ranged from 3 March 2018–29 August 2018 (Fig. 2). Ill people ranged in age from <1 to 95 years (median age: 57 years); 93/134 (69%) were female. Of the 103 ill people for whom information was available, 36 were reported to have been hospitalised. There were no reported deaths.

### Hypothesis generation

By 10 May 2018, state and local health officials were able to complete nine state-specific foodborne illness questionnaires. Common exposures reported included chicken (5/9), tomatoes (4/9) and carrots (4/9). We did not identify a common grocery store or restaurant. The NHGQ was therefore deployed on 10 May 2018 to interview all newly identified case-patients. Of 27 ill people who completed the NHGQ, no specific food exposures were found significantly more frequently among ill people in this outbreak than for respondents of the FoodNet Population Survey. Cold cereal consumption was reported in 71% (19/27) of ill people, although this was not significantly higher than the percentage of 69% from FoodNet Population Survey (*P* = 0.54) [9].

Since state-specific questionnaires and the NHGQ results did not yield a hypothesis, we conducted six open-ended interviews. During these interviews, two ill people reported that they regularly consumed a specific brand of sweetened puffed wheat cereal. When specifically asked, three of the remaining four ill people interviewed reported that they likely consumed the cereal prior to becoming sick. We reviewed previously completed NHGQ questionnaires and identified two additional ill people who reported consuming the cereal or described a product that sounded like this cereal. Given the emerging signal for sweetened puffed wheat cereal, detailed questions on cereal and related exposures were used by state and local interviewers to collect more specific information on cereal consumption. Twenty-five such interviews were ultimately completed and 18/25 (72%) responded that they had consumed or possibly consumed a single brand of sweetened puffed wheat cereal in the week prior to illness. One ill infant had not consumed the cereal but had been cared for by a family member who regularly ate the brand of cereal.

### Facility inspection

The sweetened puffed wheat cereal was manufactured and packaged in a single facility in Illinois. This manufacturing facility was owned and operated by a contractor hired by the cereal's brand owner. FDA inspected the Illinois facility in response to this outbreak from 14 June 2018–29 June 2018. Review of the manufacturer's testing records revealed that *Salmonella* had been isolated in 81 environmental samples collected between 29 September 2016 and 16 May 2018. Samples were reported to be from non-food contact surfaces, such as a flood drain and external surfaces of production equipment. *Salmonella* from environmental samples were routinely serotyped by the manufacturer's third-party lab with some samples identified as *Salmonella* Mbandaka. The manufacturing facility conducted an internal investigation for all samples yielding *Salmonella,* and corrective actions, such as cleaning and sanitising the affected areas and vector swabbing, were conducted. Vector swabbing involves taking additional environmental swabs around the positive swab area. Although 32 vector swabs were collected in response to other positive environmental samples, no documentation was available indicating that a root cause of any individual *Salmonella* finding was determined or eliminated. *Salmonella* serotypes in addition to Salmonella Mbandaka were reportedly identified during the manufacturing facility's prior environmental sampling but isolates from these other serotypes were no longer available for further analysis at the time of the FDA inspection.

FDA collected environmental swabs and a at the manufacturing facility to test for *Salmonella* spp. Three environmental swabs collected from surfaces yielded *Salmonella* Mbandaka isolates matching PFGE XbaI enzyme pattern TDRX01.0004 (outbreak strain). Three historic isolates, which were collected by the manufacturer during routine environmental monitoring and subtyped as *Salmonella* Mbandaka by the manufacturer's third-party lab, were voluntarily sent to FDA for analysis. The isolates collected by the FDA and the three isolates voluntarily shared from the manufacturer were all found to match the outbreak strain.

### Product testing

State and local officials collected a total of 5 leftover samples of the sweetened puffed wheat cereal from case-patients' homes in MT, NY, TN, TX and UT. *Salmonella* Mbandaka was isolated from three separate open samples collected from the homes of ill people in MT, NY and UT. *Salmonella* was not identified in the samples collected in TN and TX. The samples collected in MT and UT were packaged at the plant on 20 February 2018 and the sample collected by officials in NY was packaged on 21 February 2018.

Officials from the California Department of Public Health and Rhode Island Department of Health each collected unopened retail samples of the cereal. Testing of one of the samples collected in California yielded *Salmonella* Mbandaka and was packaged on 20 February 2018. This isolate was found to match the outbreak strain by PFGE. *Salmonella* was not identified in samples collected in Rhode Island.

### Whole genome sequencing

WGS analysis was completed and analysed for 125 clinical, two opened cereal samples from case-patients, three environmental swabs collected from the manufacturing facility during FDA inspection, and three historic isolates collected by the manufacturing facility prior to this outbreak. With the exception of one of the three environmental isolates collected by FDA, all isolates were considered to be highly related (0–9 SNP differences); the remaining FDA isolates were considered to be related to isolates within this main clade, though with 14–24 SNP differences from all other isolates.

### Public health action

Based upon the epidemiological investigation and review of the manufacturer's microbial testing records, which identified *Salmonella* Mbandaka isolates in the cereal production facility, the owner of the cereal brand issued a voluntary recall of all the sweetened puffed wheat cereal product within expiry on 14 June 2018. The CDC, FDA, and the cereal manufacturer utilised multiple methods to communicate the recall to the public. CDC posted four web updates during the investigation, resulting in 219 831 page views, constituting 3% of 7 343 807 page views for all multistate foodborne outbreak postings by CDC in 2018. CDC also used social media to remind consumers and retailers of the recall. Ten CDC Facebook posts about the outbreak resulted in 18 568 likes, 28 779 shares and 9838 comments. Ten CDC tweets resulted in 10 931 retweets and 10 715 URL clicks to the CDC outbreak webpage. FDA issued an International Food Safety Authorities Network (INFOSAN) Alert to inform foreign governments of international distribution of recalled product.

## Discussion

Epidemiologic and microbiologic evidence indicated that a single brand of sweetened puffed wheat cereal was the source of this outbreak. This outbreak represents the third known *Salmonella* outbreak linked to cold cereal in the United States. The first, which occurred in 1998, involved *Salmonella* Agona contamination of a toasted oats cereal which ultimately sickened over 400 people nationwide. The second outbreak occurred a decade later in 2008 and was linked to cereals produced in the same facility as the toasted oats cereal implicated in the 1998 outbreak. The outbreak strain of *Salmonella* Agona was identified in both environmental and product samples collected during the 2008 outbreak and was indistinguishable from the 1998 outbreak strain of *Salmonella* Agona by PFGE analysis. Following the 1998 outbreak, the cereal manufacturer had removed all equipment and sealed off the potentially contaminated production areas using walls to prevent further contamination of products. In 2007 an intact wall in the sealed portion of facility was disrupted for routine maintenance work, and it is hypothesised that this disruption allowed reintroduction of the bacteria [11]. Similar to the 2008 outbreak, persistent environmental contamination of the factory was also likely the source of this *Salmonella* Mbandaka outbreak presented here. Though still considered to be relatively rare, outbreaks linked to shelf-stable items are a growing concern among public health officials as a result of several recent foodborne outbreaks [5, 6, 12, 13].

It is likely that the illnesses that occurred during this outbreak would not otherwise have been connected without a robust network of molecular subtyping available through PulseNet; this allowed investigators to focus on common exposures between case-patients infected with the specific Salmonella Mbandaka outbreak strain. Nevertheless, Identification of the specific cereal implicated in this outbreak was difficult and required multiple investigational methods. Standardised questionnaires are efficient and are the most common epidemiological tool utilised in enteric disease investigations, and it is possible that the high baseline consumption of cereal within the general population did not allow for early identification of cereal as a potential vehicle. Further, there are a wide variety of cereals available within the U.S, and interviewers may not solicit specific brands from case-patients. Ill people tended to be adults over the age of 50, and although it is estimated that at least half of the U.S. population eats cereal within a given year, sweetened cereals have historically been marketed to children [9]. Evidence also suggests that cereal consumption declines with increasing age [14] This may have made investigators less likely to consider this item as a potential vehicle. The older age of individuals seen in this outbreak is particularly concerning given the increased risk of complications from *Salmonella* infections in older adults.

Illnesses due to this outbreak continued for several weeks after the initial recall despite multiple public warnings issued by CDC, FDA and the cereal manufacturer. The reason for ongoing illnesses is likely twofold. First, the cereal is sold in a variety of both physical and online retailers across the United States. Unlike fresh produce or meat products that would be removed from shelves after several days, this cereal has a long shelf life of 1 year after packaging. Lack of awareness of the recall in any one retailer or at their supplying food distributors or warehouses, could lead to ongoing sale of the contaminated cereal. Second, consumers unaware of the recall may have eaten cereal they purchased prior to the recall. Despite multiple outbreak web postings, page views for these postings were low compared to other outbreak postings that year and therefore may not have reached the target audience. This outbreak therefore demonstrates the need for recalling firms to be diligent, direct and timely when communicating about the recall to retailers and consumers. A variety of communication channels, including press releases and social media, should be utilised to disseminate the recall information broadly and inform consumers about the risk. Direct notifications to consumers and retailers about purchase or receipt of recalled product can help consumers and store employees quickly identify and take appropriate actions to mitigate any risk [15].

Recent outbreaks have reinforced growing importance of ensuring food safety standards are maintained for dried, low-moisture and other ready-to-eat (RTE) foods [3, 16]. It is estimated that processed foods, meaning items created through multiple industrial processes, account for approximately 60% of calories consumed by Americans each year [17]. Evidence suggests that foodborne pathogens, including *E. coli* and *Salmonella* species, are able to survive desiccation, and that high sugar content (such as that found in baked goods and sweetened cereals) may further support the survival of *Salmonella*. Although RTE cereals typically undergo a heat step that would kill bacterial species, there are often subsequent production steps that expose the cereal to the production environment prior to packaging that may allow introduction of bacteria onto the product. In this outbreak, all retail and opened consumer products from which the outbreak strain was isolated appear to have been manufactured and packaged on 20 February 2018 and 21 February 2018. Records collected from the plant, however, indicated that the facility had detected *Salmonella* Mbandaka in production areas beginning in 2016. Further, FDA collected *Salmonella* Mbandaka isolates highly related to the outbreak strain during inspection in June 2018. The FDA anticipates that facilities featuring robust environmental monitoring programmes will occasionally detect environmental pathogens, however, a facility's response to such findings is critical. A facility should conduct a root cause analysis and/or take prompt and aggressive corrective action to address contamination and prevent pathogens from becoming established in facility's environment [18].

The national and international distribution of shelf-stable products with long shelf lives creates the potential for large-scale, prolonged foodborne outbreaks. Stringent food safety standards within manufacturing facilities are therefore necessary at all steps of production. This investigation also highlights the difficulty in stopping outbreaks attributed to such products, as the implicated cereal product was available in a wide variety of retail settings and capable of being stored for weeks to months prior to consumption.

## Data availability statement

The data that support the findings of this study are available from the corresponding author on reasonable request.
